# Strain Sensing Coatings for Large Composite Structures Based on 2D MXene Nanoparticles

**DOI:** 10.3390/s21072378

**Published:** 2021-03-29

**Authors:** Gediminas Monastyreckis, Anastasiia Stepura, Yaryna Soyka, Hanna Maltanava, Sergey K. Poznyak, Mária Omastová, Andrey Aniskevich, Daiva Zeleniakiene

**Affiliations:** 1 Department of Mechanical Engineering, Kaunas University of Technology, Studentu St. 56, 51424 Kaunas, Lithuania; gediminas.monastyreckis@ktu.edu; 2 Polymer Institute, Slovak Academy of Sciences, Dúbravská cesta 9, 845 41 Bratislava, Slovakia; anastasiia.stepura@savba.sk (A.S.); upolyaso@savba.sk (Y.S.); maria.omastova@savba.sk (M.O.); 3 Research Institute for Physical Chemical Problems, Belarusian State University, 220030 Minsk, Belarus; maltanava@bsu.by (H.M.); poznyak@bsu.by (S.K.P.); 4 Institute for Mechanics of Materials, University of Latvia, Jelgavas str. 3, LV-1004 Riga, Latvia; andrey.aniskevich@pmi.lv

**Keywords:** MXenes, coatings, strain sensors, electrical properties, cyclic loading

## Abstract

Real-time strain monitoring of large composite structures such as wind turbine blades requires scalable, easily processable and lightweight sensors. In this study, a new type of strain-sensing coating based on 2D MXene nanoparticles was developed. A Ti_3_C_2_T_z_ MXene was prepared from Ti_3_AlC_2_ MAX phase using hydrochloric acid and lithium fluoride etching. Epoxy and glass fibre–reinforced composites were spray-coated using an MXene water solution. The morphology of the MXenes and the roughness of the substrate were characterised using optical microscopy and scanning electron microscopy. MXene coatings were first investigated under various ambient conditions. The coating experienced no significant change in electrical resistance due to temperature variation but was responsive to the 301–365 nm UV spectrum. In addition, the coating adhesion properties, electrical resistance stability over time and sensitivity to roughness were also analysed in this study. The electromechanical response of the MXene coating was investigated under tensile loading and cyclic loading conditions. The gauge factor at a strain of 4% was 10.88. After 21,650 loading cycles, the MXene coating experienced a 16.25% increase in permanent resistance, but the response to loading was more stable. This work provides novel findings on electrical resistance sensitivity to roughness and electromechanical behaviour under cyclic loading, necessary for further development of MXene-based nanocoatings. The advantages of MXene coatings for large composite structures are processability, scalability, lightweight and adhesion properties.

## 1. Introduction

In the past decade, the demand for nanosensors and electrically conductive polymer composites has grown considerably [1,2]. The tunability of properties based on nanoparticle materials and their mixtures can meet the requirements for various electronic devices and sensors. Currently, one of the largest known 2D nanoparticle families is the MXenes [3,4]. The most studied MXene particle is the titanium carbide Ti_3_C_2_T_z_, which has shown excellent mechanical and electrical properties (both in pristine form and in polymer composites) from nanoindentation measurements [5], molecular dynamics simulations [6], and finite element simulations [7,8]. Despite the relatively low van der Waals forces between 2D nanoflakes [9], pure MXene films have shown very high tensile strengths (590 MPa for a 980 nm thick film), which was obtained using large-diameter aligned flakes and the blade-casting method [10]. These properties can be influenced by different delamination methods [11,12]. During the etching and delamination process, the flake size and thickness of MXene particles can differentiate [13]. In addition, surface functional groups (T_z_), such as –O, –OH, and –F [14] (which are responsible for adhesion and wettability properties), can be modified during the process. Experimental research and finite element modelling have shown that MXenes adhere to epoxies and polymers [15,16,17]. Easily processable and scalable methods, such as spray coating [18], vacuum filtration [19], 3D printing [20] and casting [10], have shown that MXenes are also very attractive nanoparticles for coatings and thin-film applications. MXenes have already been investigated and have revealed good results for Li-ion batteries [21], organic solar cells [22], electromagnetic interference shielding [23] and supercapacitors [24].

The unique multilayered and porous structure of MXene films can cause a highly sensitive response to deformation and morphology changes. Owing to the molecular intercalation between the nanolayers, MXene films behave as very sensitive multi-gas sensors [25] and humidity sensors [26]. Furthermore, MXene aerogel foams and other layered polymer nanocomposites can be used as ultra-sensitive pressure sensors with detection limits of up to 100 Pa [27]. Sandwich-type nanolaminates and nanosensors developed from a mixture of 2D nanoflakes, nanowires, and nanospheres [28,29,30] have shown a very wide strain-sensing range owing to their enhanced electrical conductivity and flake-to-flake sliding ability. One of the most sensitive strain sensors was obtained using MXene-intercalated textile yarns. Knittable and washable MXene-coated yarns have been shown to have ultra-sensitive strain responses with a gauge factor (GF_152%_ at a strain of 152%) of up to 12,900 [31]. Another study was performed with MXene-silver nanowire coatings under tensile loading [28], where the GF_5%_ reached approximately 10. The same experiment was performed with graphene-silver nanowire coatings, where GF_5%_ reached only 2.5 [32]. Meanwhile, carbon nanotube (CNT) modified polymers [33] possessed much lower strain sensing capability, where GF_5%_ varied between 0.1 and 0.25.

The electromechanical mechanism of nanoparticle-based strain sensors is well explained by an analytical approach [34]. Surface topography images also provide in-depth details on how the structure of the nanocoating behaves under a wide range of deformations (0–60%) [32]. It was also found that the electrical sensitivity was enhanced by electron tunnelling effects [35], which mostly occur when the distance between the nanoparticles is less than 3 nm. An analytical approach describing the tunnelling effect explains how electric resistance can change under nanoscale deformations [36].

Traditional sensors, such as strain gauges, accelerometers, piezoelectric transducers, and fibre optic cables, have been used for wind turbine monitoring [37]. These sensors typically experience adhesion problems and application difficulties, requiring precise sensor calibration and complex signal processing [38]. Despite this, fibre Bragg grating (FBG) optical sensors are widely used due to their most accurate strain measurements. FBG sensors are is still being improved by materials such as magnetostrictive Terfenol-D (Tb_0.3_Dy_0.7_Fe_1.92_), with whom the sensitivity of the system is improved by a factor of 4 [39].

Until now, MXenes have not been investigated as strain-sensing coatings for fibre-reinforced polymer composites. The aim of this study is to develop an easily processable and scalable MXene coating that can detect the low strain values typical for fibre-reinforced composites. The main tasks were to characterise the morphology and uniformity of the coating, investigate the response to environmental conditions (such as temperature and UV irradiation), analyse the surface roughness influence on sensing performance and study the sensing behaviour under tensile loading and cyclic loading conditions. The strain sensing ability of the MXene coating was based on the change in electrical resistance. Simple spray-coating methods and electrical resistance monitoring systems were a priority during the experiments.

## 2. Materials and Methods

### 2.1. Materials

Ti_3_C_2_T_z_ MXenes were prepared from Ti_3_AlC_2_ MAX phase with a particle size of <40 µm and purity of 98 wt.% (MRC, Kiev, Ukraine) using a previously documented method [12]. The etching solvents used were hydrochloric acid (37 wt.%, Merck, Darmstadt, Germany) and lithium fluoride (>99 wt.%, Sigma Aldrich, Munich, Germany). LiF was mixed with HCl to generate HF in the system. The MAX phase was slowly added and stirred for 24 h. The multilayer MXene sediment was further delaminated using 99 wt.% LiCl (Sigma Aldrich, Munich, Germany). The resulting solution was centrifuged 10–15 times at 3500 rpm and washed with deionised water until the pH of the supernatant reached 6.5. The concentration of the delaminated MXenes in the supernatant was 0.335 mg/mL, but it was further centrifuged until the concentration reached 3.3 mg/mL to be more suitable for the spraying process.

The thermosetting epoxy resin Bisphenol F-epichlorohydrin (Biresin^®^ CR122, Sika AG, Baar, Switzerland) and an amine curing agent (Biresin^®^ CH122-5) were mixed at a ratio of 10:3. Epoxy tensile specimens (ISO-527-2-5A) were cast in silicone moulds. Glass fibre–reinforced polymer (GFRP) tensile specimens and sandwich-type GFRP specimens were prepared for adhesion tests by hand-layup and vacuum bagging methods. GFRP tensile specimens (15 cm × 1.5 cm) were made of 5 plies of twill-weave 2/2 (163 g/m^2^) Interglas 92110 (Porcher Industries, Erbach, Germany), and adhesion specimens (14 cm × 2.5 cm) were made of a total of 8 plies and 4 mm thick AIREX C70.75 foam (Airex AG, Sins, Switzerland). All specimens were cured at room temperature for 24 h and post-cured in a convection oven for 5 h at 100 °C. Additionally, epoxy samples were roughened in the Y direction (parallel to loading), X direction (perpendicular to loading), and YX direction (roughened in both directions) using P280 sandpaper (52.2 µm average particle diameter). All samples were plasma-treated and spray-coated with a water-based MXene solution, as described below.

### 2.2. Preparation of MXene Coating

**Figure 1:**
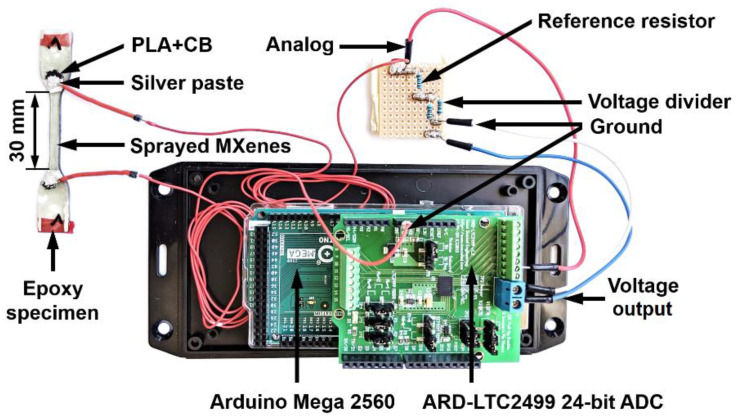
MXene-coated epoxy specimen and electrical resistance measurement system.

To enhance the adhesion between the epoxy substrate and MXenes, a surface plasma treatment was performed using a Zepto Diener low-pressure plasma cleaner (Diener Electronic GmbH & Co. KG, Ebhausen, Germany) and a K1050X RF plasma cleaner (Quorum Technologies, Laughton, UK). The samples were treated under vacuum with no additional gases for 3 min with 100 W power at 40 kHz frequency. The water contact angle was reduced from 68.7° (for pristine epoxy) to 25.3° (for the modified epoxy surface). Another treatment was performed in a vacuum with an oxygen and argon enriched atmosphere for 3 min with 100 W at 13.56 MHz. The contact angle was reduced to 20.1°. After the epoxy surface plasma treatment, water-based MXenes were sprayed using a Sparmax HB-040 airbrush with a 0.4 mm diameter nozzle and a Sparmax DC-25X 2.07 bar compressor with 0.1 mL/s paint yield (Anest Iwata Sparmax Co., Taipei, Taiwan). The MXene coating was applied by spraying for 10 s at a distance of 15 cm, which naturally dried over 5–10 s at room temperature. Copper wires were directly soldered onto the MXene films using the commercially available electrically conductive polymer Protopasta (Protoplant, Inc., Vancouver, WA, USA). This polymer is made from a mixture of polylactic acid (PLA) (4043D PLA, Natureworks, Minnetonka, MN, USA) and carbon black (CB). The volume resistivity of the polymer is 30 Ωcm, and the melting point is 155 °C. The soldering needle temperatures were set to 200 °C, similar to the 3D printer nozzle temperatures. The soldered wires were additionally covered with a thin layer of silver paste. The distance between the silver paste contacts for both the epoxy and GFRP samples was 30 mm. The epoxy specimen sprayed with MXenes and a monitoring system (described in Section 2.3) are presented in Figure 1.

### 2.3. Characterisation and Testing Equipment

X-ray photoelectron spectroscopy characterisation of the same Ti_3_AlC_2_ MAX phase and Ti_3_C_2_T_z_ MXenes was previously reported by Zukiene et al. [16]. The thickness and topography of the MXene coatings were characterised using scanning electron microscopy (SEM) (JEOL JSM 6610, JEOL Ltd., Tokyo, Japan). Gold-coated samples were measured under high-vacuum mode with an accelerating voltage of 15 kV.

The homogeneity of the MXene coating after plasma treatment and the roughness of the substrate were investigated using a Leica DVM6 optical microscope (Leica Microsystems, Wetzlar, Germany). 3D optical topographical scanning was performed using the image rendering software LAS X (Leica Microsystems), based on the stacking of 2D images in the Z direction.

Tensile and fatigue tests were performed using Tinius Olsen H25 KT (Tinius Olsen, Salfords, UK) and Instron ElectroPuls E10000T (Instron, Norwood, MA, USA) equipment, respectively. Deformations in the longitudinal direction between four strain markings (2.5 cm distance) and transversal deformations between six strain markings (4 mm distance), were measured using Manta G-146B visual extensometer (Allied Vision Technologies, Stadtroda, Germany). Peeling on epoxy samples (5 cm × 0.5 cm) covered with the MXene coating was investigated using simple household adhesive tape. Pull-out tests of MXene-coated sandwich-type GFRP specimens were performed using an Adheometr PM 420/63 under ISO-4624 standards. For the UV light absorbance experiment, a UV DRT230 lamp with a 301–365 nm emission wavelength and a surface power density of 3–7 mW/cm^2^ was used. Experiments were performed on an MXene-coated sandwich-type GFRP specimen (13.5 cm × 2.5 cm). The response of the MXene coating to direct sunlight was measured under a clear sky with a solar elevation angle of 54° (Riga, Latvia).

The temperature and initial electrical resistance values were monitored using a Fluke 287 True-RMS logging multimeter (Fluke Corporation, Everett, WA, USA). The temperature of MXenes was measured using a temperature probe soldered to aluminium foil (0.3 mm × 25 × 30 mm), which was pressed to a 0.2 mm thick protective epoxy layer on top of the MXene coating. An MXene coating area of the same size (25 mm × 30 mm) was heated with 10–90 V (DC) using AX-12001DBL external power supply (Transfer Multisort Elektronik, Łódź, Poland). For the tensile-tensile fatigue tests, electrical resistance values were measured using Arduino Mega 2560 microcontroller (Figure 1) and ATmega2560 microchip (Arduino, Turin, Italy). Additionally, a 24-bit analogue-to-digital converter (ARD-LTC2499, Iowa Scaled Engineering, Elbert, CO, USA) with a precise 4.096 V output voltage and 7 Hz measurement frequency was used for high-accuracy electrical resistance measurements. The electrical resistance monitoring during mechanical loading was based on the voltage difference between a single reference resistor and the MXene coating.

## 3. Results

### 3.1. MXene Coating Topography

**Figure 2:**
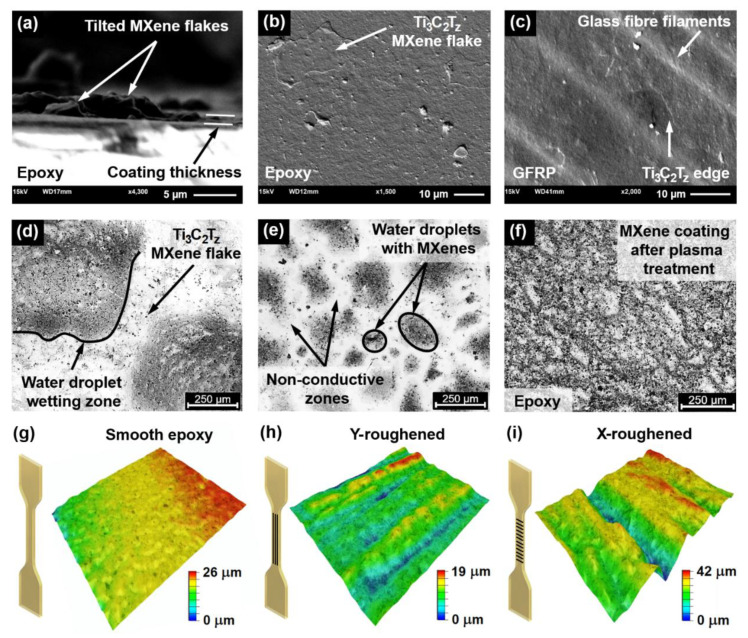
Scanning electron microscopy images of the sprayed MXenes: (**a**) thickness of the coating; (**b**) topography of MXenes on epoxy, and (**c**) glass fibre–reinforced polymer samples. Optical microscopy images of the MXene coatings: (**d**) sprayed for 2 s at 3 cm; (**e**) sprayed for 10 s at 15 cm; and (**f**) sprayed for 10 s at 15 cm on a plasma-treated epoxy sample. Optical topography images of epoxy samples that were (**g**) smooth, (**h**) roughened in the Y direction, and (**i**) roughened in the X direction.

The morphology of the MXene flakes and the coating was studied using SEM. The average thickness of the coating was approximately 1 µm, as evident in the cross-sectional view of the fractured epoxy sample presented in Figure 2a. A plasma-treated smooth epoxy sample coated with MXenes is presented in Figure 2b (top view). A Ti_3_C_2_T_z_ flake with a size of 20 µm × 10 µm can be seen, while sizes, ranging from 1 to 5 µm are the most common, as it was reported in previous SEM analysis [16]. The MXene-coated GFRP sample is shown in Figure 2c (top view), in which glass fibre filament markings and MXene flake edges can be seen.

Owing to the optical transparency of the epoxy and MXenes [40], the overall quality of the coating was analysed using an optical microscope with an external light source. First, small water droplets containing higher (darker areas) and lower (lighter areas) concentrations of MXene flakes were observed in optical microscopy images (Figure 2d). The primary reason for this effect was the hydrophobic epoxy surface. In addition, the size of these droplets and coating uniformity depended on the spraying time and nozzle distance. For example, when sprayed for 2 s at 3 cm from the surface, water droplets were 250–500 µm in size (Figure 2d), while spraying for 10 s at 15 cm reduced the droplet size to 50–250 µm (Figure 2e). Plasma treatment was performed to enhance the hydrophilic properties of the epoxy surface, which increased the conductivity and uniformity of the coating (Figure 2f). The sensitivity of strain sensors based on nanoparticles depends on the morphology of the substrate surface [41]. To investigate this effect, epoxy tensile specimens were roughened in the Y, X, and YX directions. 3D optical topography images (600 µm × 450 µm) are presented in Figure 2g–i, and detailed average roughness values are presented in Section 3.4

### 3.2. MXene Coating Adhesion and Stability

**Figure 3:**
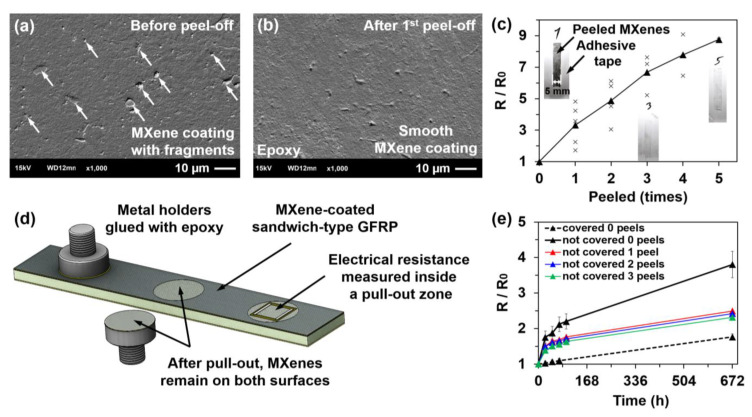
Scanning electron microscopyimages of the MXene coating (**a**) before peeling and (**b**) after the first peel-off attempt; (**c**) electrical resistance change during five peel-off attempts; (**d**) MXene coating pull-out test; and (**e**) electrical resistance change over a 4 week period.

The adhesion to epoxy and the stability of the MXene coatings were investigated using SEM images and electrical resistance changes during every peel-off attempt with adhesive tape. An SEM image of the sample before peeling is presented in Figure 3a, where an MXene coating with small fragments can be seen. After the first peel-off, the surface of the MXene coating became smooth and no fragments, rupture spots, or debonding from the epoxy was observed (Figure 3b). However, the relative electrical resistance increased with every peel-off attempt (Figure 3c). After the first peel-off, the relative resistance increased 3.2 times, and it increased 8.6 times after the fifth. After five peeling attempts, no damage to the coating was observed, which suggests that adhesion between the MXenes and the epoxy was strong, as mentioned in several previous studies [15,16,17]. To extend the adhesion experiments, an MXene coating pull-out test was performed (Figure 3d). The Ti_3_C_2_T_z_ water solution was sprayed on a more rigid sandwich-type GFRP composite. In total, six pull-out attempts were performed on two GFRP specimens, and an average pull-out stress of 2.14 MPa was obtained. Despite the pull-out, the inner area retained MXene flakes, whose electrical resistance increased roughly 19 times compared to the initial values (Figure 3d). Therefore, the actual pull-out stress (2.14 MPa) was obtained between MXene-MXene flakes in the perpendicular direction. This stress is roughly 10 times lower than the tensile strength of pure MXene-MXene films [42,43]. These results are very important for the further development of strain sensing MXene coatings.

Coatings based on Ti_3_C_2_T_z_ MXenes tend to degrade by their oxidation to TiO_2_ in various exploitation conditions [44]. In this article, the MXene coating stability was investigated over a four-week period at room temperature (Figure 3e). The relative electrical resistance over four weeks of a sample peeled one time was 2.49, while that of a non-peeled sample was 3.80. This suggests that a coating with a smoother surface will oxidise slower owing to the smaller nanoparticle area exposed to air. The best electrical resistance stability of the MXene coatings was achieved with a protective epoxy layer. In comparison, the electrical resistance of the sample without a covering layer increased the fastest, and the resistance after four weeks was 3.7 times higher than that of the covered sample. Such oxidation rates of MXenes do not satisfy application requirements for sensors, where on the contrary, MAX phase-based sensors show long-term stability [45]. Therefore, the focus still remains on the development of new oxidation preventing methods such as hydrogen annealing [46] and L-ascorbic acid treatment [47], which have already shown positive effects.

### 3.3. MXene Coating UV and Temperature Response

**Figure 4:**
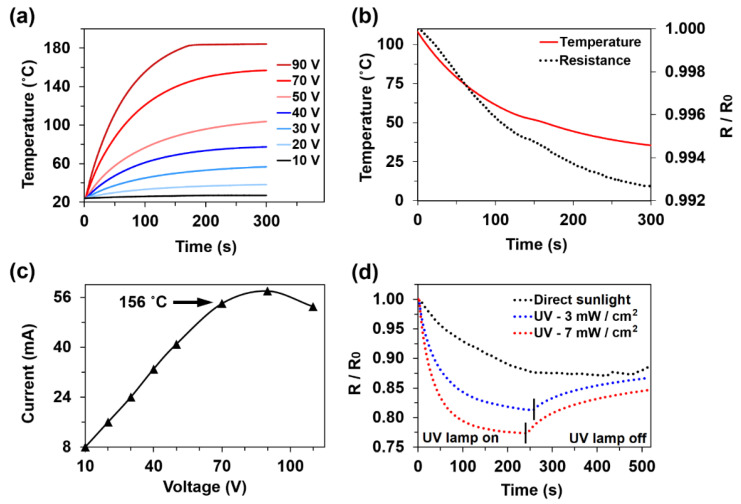
(**a**) Increase in the MXene coating temperature under different applied voltages; (**b**) change in the electrical resistance during the natural cooling process of the sample; (**c**) response of the electric current to applied voltage; and (**d**) relative electrical resistance response of the MXene coating to direct sunlight and UV irradiation.

Experiments were performed to understand deviations in the electrical signal from the MXene coatings due to ambient conditions, such as direct sunlight and temperature. Several studies have shown that MXene films can experience high heating temperatures (114 °C) under an applied voltage of 6 V [48]. In this study, coating temperatures under 10–90 V were investigated. When 10 V was applied, the temperature of the MXenes only increased from 24 to 27 °C (Figure 4a) owing to the high resistance of the coating (0.91 kΩ). In comparison, at 50 V, the coating temperature increased to 103.8 °C, and at 90 V, the coating temperatures plateaued at 183.7 °C, which is close to the epoxy melting temperature. Detailed results of the temperature response under different voltages are presented in Figure 4a. The electrical resistance was measured after 50 V was applied and then removed, and the coating cooled naturally from 103.8 to 35.6 °C over 300 s (Figure 4b). During this process, the electrical resistance decreased to 0.8%. The electric current was also monitored during the heating process with an applied voltage (Figure 4c). When the temperature of the coating reached 156 °C (70 V), the resistance of the coating began to increase, and the electric current dropped. This occurred because of the softened epoxy surface, which resulted in a change in the morphology and a rearrangement of the MXene flakes. In addition, no degradation of Ti_3_C_2_T_z_ could occur, according to previous thermogravimetric analysis [49], which shows that the MXene is thermally stable and loses only 4–7% of its weight at 700 °C.

As described in several articles [50], MXene and other nanoparticles, such as graphene, exhibit high absorbance in the 200–400 nm UV spectrum. Therefore, the electrical resistance response of the MXene coating to direct sunlight was measured. After 5 min under direct sunlight, the electrical resistance of the coating decreased by 12.5% (Figure 4d). Depending on the UV lamp irradiance intensity, the response of the coating reached a steady state after 4 min, and the electrical resistance decreased by 22.7% at 7 mW/cm^2^ (35 °C specimen temperature). These results are important because they help to avoid electrical signal disturbance due to ambient conditions. Therefore, tensile and fatigue tests were performed without daylight, and the temperatures of the samples were monitored during the testing.

### 3.4. Tensile Tests of MXene-Coated Epoxy Specimens

In this experiment, the electromechanical behaviour of MXene coatings under tensile loading was monitored on smooth and roughened epoxy samples. The average roughness values in the X and Y directions, initial resistance values (R_0_), resistance values at the sample breaking point (R_b_), and gauge factors at strains of 1% (GF_1%_) and 4% (GF_4%_) of 4 samples with different roughness values (0, Y, X, and YX) are presented in Table 1.

**Figure 5:**
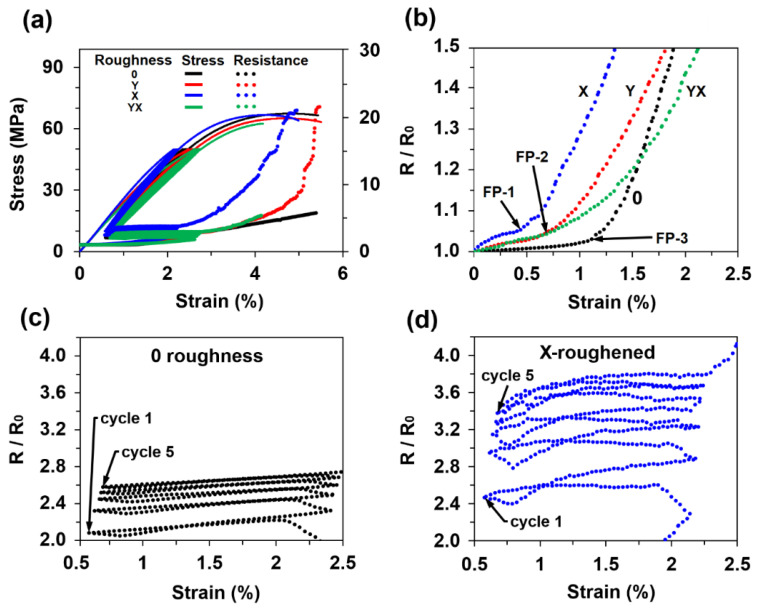
Results from tensile tests with five initial loading cycles on smooth and roughened MXene-coated epoxy samples: (**a**) mechanical response and relative electrical resistance measurements of four samples with different roughening; (**b**) magnified region showing the electrical resistance at low strains; and MXene resistance response under five loading cycles with 10–50 MPa amplitudes on (**c**) a smooth epoxy sample and (**d**) an X-roughened sample.

The relative electrical resistance response of the MXene coatings to 5 initial loading cycles, with amplitudes of 10 to 50 MPa, are presented in Figure 5a. A considerable difference in initial resistance was obtained between the smooth and X-roughened samples (8.48 kΩ versus 674 kΩ, respectively). In addition, the X-roughened sample with a roughness of 2.54 µm had the highest GF_1%_ of 1.29, while the smooth sample with an average roughness of 0.56 µm showed only 1.02 (Figure 5b). Notable changes in the relative resistance curves for all the samples appeared between strains of 0.5% and 1.1% (Figure 5b). Previous research on pure MXene films under tensile conditions yielded similar fracture strain values, which are marked in the figure as MXene fracture points (FP): FP-1 [48], FP-2 [49], and FP-3 [42,43]. These results suggest that MXene flakes began to debond from one another as the resistance began to increase drastically. Another important comparison is presented in Figure 5c,d, which shows that the resistance behaviour on the smooth epoxy substrate was stable during cycling, while on the rough substrate, the values were more chaotic. These results help to understand the electrical signal response under different strain values as well as to illuminate how even a small difference in roughness can have a substantial impact on the coating sensitivity. For instance, MXene-silver nanowire coatings reached GF_5%_ of 10 [28], while in this research, depending on the roughness, GF_4%_ was obtained in the range of 4.17–10.88.

### 3.5. Fatigue Tests of MXene-Coated Epoxy Specimens

**Figure 6:**
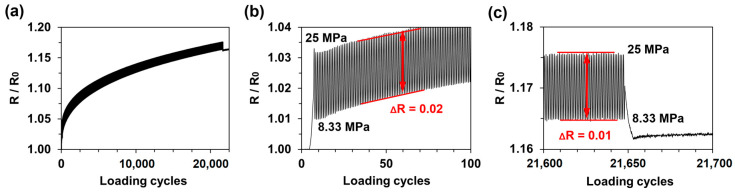
Fatigue test results of a smooth MXene-coated epoxy sample: (**a**) relative resistance during fatigue testing with an 8.33–25 MPa amplitude, and magnified regions showing (**b**) the first 100 cycles and (**c**) the last 50 cycles.

After the initial tensile tests, tensile-tensile fatigue tests were performed with smooth MXene-coated epoxy specimens. Loading cycles were performed in the elastic region of the specimens at 0.5 Hz and an 8.33–25 MPa loading amplitude (0.31–0.97% strain). The specimen temperatures were monitored throughout the tests and were maintained at 25 °C. The change in electrical resistance over 21,650 cycles can be seen in Figure 6a. The electrical resistance amplitude (Δ*R*) at the beginning of the fatigue test can be seen in the magnified region showing the first 100 cycles (Figure 6b), where Δ*R* was approximately 2%. It is important to note that the electrical resistance permanently increased after every cycle, and after 21,650 loading cycles, the MXene coating experienced an irreversible resistance increase of 16.25%. The last 50 cycles are shown in Figure 6c, where Δ*R* decreased from 2% to 1%, which indicates that the coating became less sensitive. However, the important result was that the rate of increase of the irreversible resistance became much lower, i.e., the MXene coating electrical signal became more stable to mechanical loading. The results of the variation in the electrical resistance of the MXene coating over twenty-one thousand cycles under the same loading conditions are important for the development of signal processing algorithms and further coating improvements.

### 3.6. Fatigue Tests of MXene-Coated GFRP Specimens

The tensile-tensile fatigue response of the MXene coating was further investigated using 0° and 45° fibre angle GFRP specimens. The samples were first tested until fracture, and tensile strengths of 393.3 and 120.3 MPa and fracture strains of 2.86% and 6.98% were obtained for the 0° and 45° fibre specimens, respectively. The GF_2%_ of the 0° specimen was 1.72, and that of the 45° specimen was 1.08. The fatigue tests were performed at 0.5 Hz with a variable loading amplitude. Detailed loading properties are presented in Table 2 and labelled according to the loading steps (force, stress, and strain).

**Figure 7:**
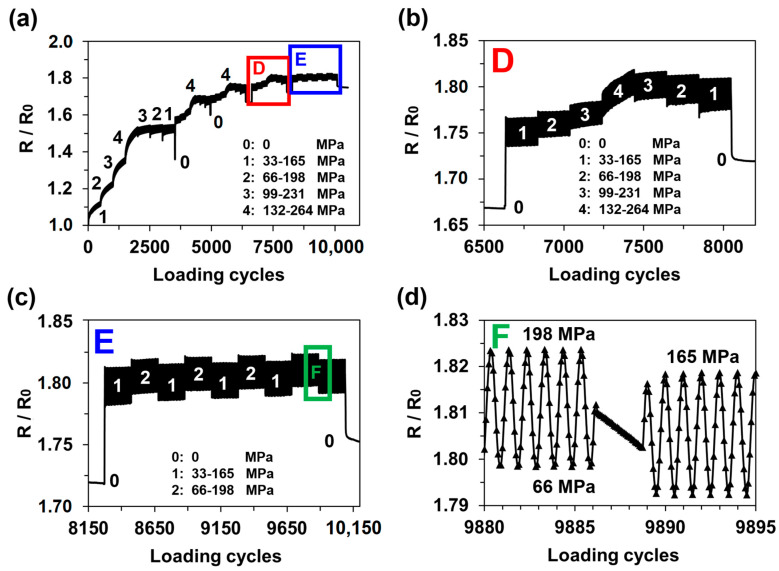
Fatigue test results for MXene-coated 0° glass fibre–reinforced polymer specimen: (**a**) relative electrical resistance response under 10000 loading cycles with varying amplitude; (**b**) magnified region showing the loading step series 0-1-2-3-4-3-2-1-0; (**c**) magnified region showing the loading step series 1-2-1; and (**d**) magnified region showing the loading step change from 2 to 1.

The fatigue tests of the MXene-coated 0° and 45° fibre angle GFRP specimens showed similar strain sensing tendencies; therefore, only the results of the 0° specimen under different loading steps are presented in Figure 7a. In total, four loading step sequences (0-1-2-3-4-3-2-1-0) were applied. It can be observed that the MXene coating sensitivity to strain becomes more stable with repeated loading steps. First, the irreversible resistance increased at every loading step, but after several thousand cycles, the permanent resistance only increased during the fourth step, which had a high loading amplitude (Figure 7b). After four series of loading step sequences, the sample was loaded with low amplitude steps (1-2-1) (Figure 7c). During this stage, the strain sensing of the MXene coating was stable, and it was possible to identify the exact tensile stress (and strain) according to the relative resistance values. A magnified region showing the electrical resistance is presented in Figure 7d. The accuracy obtained using only a single reference resistor scheme with no additional filtering or amplification of the signal demonstrate that the MXenes are strain-sensitive nanoparticles. Although, the resistance amplitude at small strain regions (0.21–1.07%) is not very high (*ΔR* = 2.7%), when compared to CNT intercalated MXene sensors (*ΔR* = 5.5% at 0.2–1% strain) [29]. Pure MXene coatings can still be competitive due to their processability, scalability and good adhesion properties when compared to MXene-CNT nanocoatings.

## 4. Conclusions

In this study, a comprehensive electromechanical investigation of MXene-coated epoxy and GFRP specimens was performed. Ti_3_C_2_T_z_ was synthesised using HCl and LiF etching. A water-based MXene solution was sprayed onto plasma-treated epoxy and GFRP samples.

Before the tensile experiments, the response of MXene coatings to heating and UV irradiation was analysed. The MXene coating experienced no significant increase in electrical resistance until the epoxy substrate began to degrade at 156 °C. After 5 min under direct sunlight, the coating resistance decreased by 12.5% and reached a plateau. Under 301–365 nm UV light, the resistance decreased by 22.7%.

To investigate the strain sensitivity behaviour of the MXene coating, epoxy samples were roughened in directions perpendicular and parallel to the tensile loading. The surface roughness was measured using 3D optical microscopy scanning, and the morphology of the MXenes was characterised using SEM. The MXene strain sensitivity was primarily dependant on the perpendicular roughness, where the gauge factor at 4% strain was 10.88, while that of the parallel roughness was 4.81.

The increase in the electrical resistance of the MXene coatings over time, durability to peeling, and MXene adhesion to the epoxy surface was also studied. The pull-out stress obtained between MXene-MXene particles in the perpendicular direction was 2.14 MPa.

The main focus of this study was to investigate the electrical resistance response of MXene-coated epoxy and GFRP samples under tensile-tensile fatigue loading. After 21,650 loading cycles at a constant loading amplitude, the MXene coating on the epoxy experienced a 16.25% increase in permanent resistance. GFRP samples were tested under varying amplitudes with high tensile loads. After 10,000 cycles, the electrical resistance of the MXene coating permanently increased by 1.8 times, but the response to loading was more stable and equal to 2.7% resistance change at a strain region of 0.21–1.07%.

These results demonstrate that MXenes are a viable material for ultra-thin, scalable, and easily processed strain-sensing coatings for large fibre-reinforced composite structures.

## Figures and Tables

**Table 1:** Electrical resistance response of the MXene coatings during tensile tests.

Roughening Direction	Average Roughness in X/Y Directions (µm)	R_0_ (kΩ)	R_b_ (kΩ)	GF_1%_	GF_4%_
0	0.56	8.48	49.3	1.02	4.17
Y	0.68/2.31	48.3	1049	1.12	4.81
X	2.54/1.32	674	14924	1.29	10.88
YX	1.67/1.63	647	3604	1.08	5.01

**Table 2:** Fatigue loading steps: loading amplitude, tensile stress, and tensile strain.

Loading Step	Loading Amplitude (kN)	Tensile Stress (MPa)	Tensile Strain (%)
0	0	0	0
1	0.25–1.25	33–165	0.21–1.07
2	0.50–1.50	66–198	0.42–1.33
3	0.75–1.75	99–231	0.63–1.59
4	1.00–2.00	132–264	0.85–1.86

## Data Availability

Not applicable.
